# Identification of Lower-Limb Motor Tasks via Brain–Computer Interfaces: A Topical Overview

**DOI:** 10.3390/s22052028

**Published:** 2022-03-04

**Authors:** Víctor Asanza, Enrique Peláez, Francis Loayza, Leandro L. Lorente-Leyva, Diego H. Peluffo-Ordóñez

**Affiliations:** 1 Facultad de Ingeniería en Electricidad y Computación, Escuela Superior Politécnica del Litoral (ESPOL), Campus Gustavo Galindo km 30.5 Vía Perimetral, Guayaquil P.O. Box 09-01-5863, Ecuador; epelaez@espol.edu.ec; 2 Neuroimaging and Bioengineering Laboratory (LNB), Facultad de Ingeniería en Mecánica y Ciencias de la Producción, Escuela Superior Politécnica del Litoral (ESPOL), Campus Gustavo Galindo km 30.5 Vía Perimetral, Guayaquil P.O. Box 09-01-5863, Ecuador; floayza@espol.edu.ec; 3 Centro de Posgrado, Universidad Politécnica Estatal del Carchi, Tulcán 040101, Ecuador; leandro.lorente@upec.edu.ec; 4 Faculty of Engineering, Corporación Universitaria Autónoma de Nariño, Pasto 520001, Colombia; peluffo.diego@um6p.ma; 5 Modeling, Simulation and Data Analysis (MSDA) Research Program, Mohammed VI Polytechnic University, Ben Guerir 43150, Morocco

**Keywords:** brain–computer interfaces (BCI), electroencephalogram (EEG), lower limb, pattern recognition (PR), topical overview

## Abstract

Recent engineering and neuroscience applications have led to the development of brain–computer interface (BCI) systems that improve the quality of life of people with motor disabilities. In the same area, a significant number of studies have been conducted in identifying or classifying upper-limb movement intentions. On the contrary, few works have been concerned with movement intention identification for lower limbs. Notwithstanding, lower-limb neurorehabilitation is a major topic in medical settings, as some people suffer from mobility problems in their lower limbs, such as those diagnosed with neurodegenerative disorders, such as multiple sclerosis, and people with hemiplegia or quadriplegia. Particularly, the conventional pattern recognition (PR) systems are one of the most suitable computational tools for electroencephalography (EEG) signal analysis as the explicit knowledge of the features involved in the PR process itself is crucial for both improving signal classification performance and providing more interpretability. In this regard, there is a real need for outline and comparative studies gathering benchmark and state-of-art PR techniques that allow for a deeper understanding thereof and a proper selection of a specific technique. This study conducted a topical overview of specialized papers covering lower-limb motor task identification through PR-based BCI/EEG signal analysis systems. To do so, we first established search terms and inclusion and exclusion criteria to find the most relevant papers on the subject. As a result, we identified the 22 most relevant papers. Next, we reviewed their experimental methodologies for recording EEG signals during the execution of lower limb tasks. In addition, we review the algorithms used in the preprocessing, feature extraction, and classification stages. Finally, we compared all the algorithms and determined which of them are the most suitable in terms of accuracy.

## 1. Introduction

EEG signal detection has shown excellent results in medical applications, including the early detection of neurological disorders [1], such as Alzheimer’s disease [2]. In addition, the detection of movement intentions by processing EEG signals is the basis of the non-invasive brain–computer interface (BCI). This application benefits people with motor disabilities by allowing them to control robotic prostheses [3], biomechanical assistive orthoses [4], lower-limb robotic exoskeletons [5], and home automation devices [6,7].

Biomedical EEG signals acquired from the human scalp are used to record the activity of the cerebral cortex in the order of micro-volts [8]. These applications seek to improve the quality of life of people with motor disabilities but require wearables or medical equipment capable of acquiring EEG signals with a low signal-to-noise ratio [9]. EEG signals are recorded in the time domain and—because of being bioelectrical-type—are very sensitive to the noise produced mainly by the relative movement between the surface electrodes and the scalp, skin sweating, heartbeat, blinking, and harmonics of the electrical network [10].

Applications based on movement intention detection with unsupervised [11] and supervised [12] Machine Learning (ML) algorithms use EEG signals. Due to the low signal-to-noise ratio of these signals, a preprocessing and feature extraction stage is required [13]. However, some studies, such as those carried out by Garcia-Moreno, use Deep Learning algorithms for the detection of movement intentions [14]. These methods are highly used nowadays because they do not require a preprocessing or feature extraction stage.

There is an unprecedented growth of devices to detect brain activity while performing motor tasks [15]. In addition, medical studies reveal that EEG signals can be used to detect movement intentions in people suffering from neurological disorders such as epilepsy and autism spectrum disorder [16], Alzheimer’s disease [17,18,19,20], and Parkinson’s disorder (PD) [21,22,23,24]. Consequently, people with motor disabilities may control assistive devices or prostheses using noninvasive EEG sensors [25].

Particularly, in the context of BCI- or EEG-based motor task identification, a substantial number of research papers have focused on the upper-limb analysis while only a few have been devoted to lower-limb-related applications.

Furthermore, to the best of the authors’ knowledge and in light of the here-obtained findings, the potential of lower-limb-related BCI (EEG signal analysis) has not been comprehensively exploited, at least at the level of the design of a conventional pattern recognition (PR) system [26], which typically includes the following building blocks: data acquisition, preprocessing, representation (e.g., characterization and/or feature extraction), and generalization (e.g., classification, clustering, and/or regression). Conventional PR alternatives are preferred in biomedical settings as they work on the basis of a feature set defined from a proper characterization step, which not only feed the subsequent classifiers but naturally provide interpretability. In addition, classification accuracy can be improved when using more expressive features [27]. That said, feature extraction is of great interest in EEG signal analysis, and thus, this work gives special attention to it.

In this sense, this paper presents a specialized review aimed at reviewing scientific papers focused on BCI-driven detection of lower-limb movement intentions. Methodologically speaking, the review is outlined as a topical overview following the terminology presented in [28]. The focus of our study is on papers addressing the motor imagery paradigm (as defined in [29]) and involving PR stages. Furthermore, the data acquisition methodologies are studied. To do so, the review process is framed within the use of PR building blocks as follows: Firstly, we explore the experimental methodologies used for data acquisition. Secondly, we identify the preprocessing and feature extraction techniques. Finally, we compare the classification algorithms.

**Figure 1:**
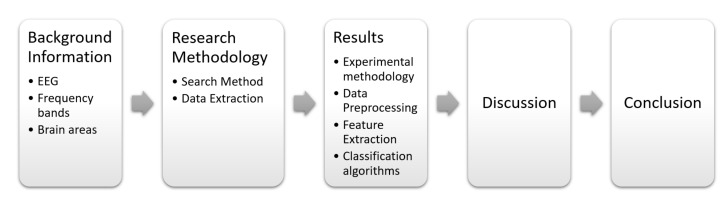
Structure of this study. It includes: A background on brain and electroencephalogram fundamentals; the proposed research methodology for searching and data extraction steps; results for the experimental methodology and the pattern recognition stages; discussion about the findings and concluding remarks.

The remaining of the manuscript is organized as follows: Section 2 briefly presents some remarkable related works to both provide a context and highlight the gap that this work is intended to bridge. Section 3 provides background information on the brain areas and the EEG signals with their different frequency bands. Section 4 describes the research methodology of searching the scientific papers in different databases. Section 5 draws the results and elaborates on answering the research question. Section 6 presents the discussion of the results obtained. Finally, Section 7 gathers the final and concluding remarks of this work. Figure 1 depicts the structure of this study.

## 2. Related Works

A large body of work on detecting movement intentions for the upper limbs based on EEG signals has led to the development of BCI systems [30,31,32,33]. Some works [34,35] even use visual stimuli based on SSVEP EEG to detect the motor intention of the subjects. Nonetheless, such works are not considered in this study as their methodologies are limited to the detection of the frequencies of the visual stimuli and make no use of the cortical activity generated during the execution of motor or motor imagery tasks. Another related work [36] presents a brain-controlled lower limb exoskeleton for rhesus macaques, which was unmistakably discarded since we are solely interested in motor activity in the cerebral cortex of humans. This application improves the quality of life of people with motor disabilities by giving them the ability to control assistive devices and active prostheses. However, the detection of movement intentions for the lower limbs has recently gained more attention from the scientific community. The number of publications on this research topic has grown in just the last three years.

As a remarkable, related work, scientific literature reports the study by Lennon et al. [37] submitted on December 2019 and published on June 2020. Such a work reviews studies about robotic gait devices interfaces for stroke rehabilitation and explores both upper-limb- and lower-limb-related signals. It covers approaches based on EEG and electromiographic signals (both individually and jointly) in a wider, exhaustive manner. Thus, due to the recent major advances in both electronic device design as well as computational and artificial intelligence techniques, an up-to-date, specialized overview is needed.

## 3. Background Information

### 3.1. Electroencephalography

The brain is responsible for leading advanced neural activities such as learning, language, memory, and intelligence in the central nervous system. As the brain works, neurons create bioelectricity, which, in turn, generates voltage fluctuations [38]. These fluctuations can be amplified and recorded, thanks to the development of electronics, using an electroencephalograph.

Biomedical EEG electrodes measure electric potentials in the scalp; those signals represent neuronal activities corresponding to each area of the brain [38]. One of the most well-known applications of EEG signals is BCIs, which make people with motor disabilities and residual cortical activity able to interact with robotic prostheses [39].

The experimental procedures to develop algorithms for analyzing and interpreting EEG brain activity are based on measuring motor tasks or motor imagery activity. These electrical signals are acquired in the time domain from the scalp with a magnitude in the order of microvolts (uV) [40,41,42].

To the best of our knowledge, techniques for detecting cortical motor activity corresponding to the lower limbs have not been widely explored. Such electric potentials are difficult to assess due to their origin in deep locations of the brain, as the central motor gyro, located in the inner side of the longitudinal fissure of the brain [43]. Thus, some topographic visualization techniques based on EEG data of cortical motor activity are mainly focused on the upper limbs [44,45]. For example, Yoon Kyum Shin et al. [44] demonstrate the difference in the prefrontal cortex when performing motor activities with the hands and when just imagining moving them. In the present work, we included the analysis of motor and imaginary movement of the legs and feet.

### 3.2. Frequency Bands

Cortical activity is represented in the behavior of EEG signals in a frequency range from under 4 to 140 Hz. This range includes the following frequency bands:

- *Delta wave*: frequencies below 4 Hz. It has been detected in infants or adults during deep sleep [46];
- *Theta wave*: frequencies between 4 and 7 Hz. It is detected in youngsters and adults in stages of drowsiness [47].
- *Alpha wave*: frequencies between 8 and 12 Hz. It is detected in young people and adults during low brain activity or rest [46].
- *Mu wave*: 7.5–12.5 (Performs a motor action):
  - Unlike the alpha wave, which occurs at a similar frequency over the resting visual cortex at the back of the scalp, the mu wave is found over the motor cortex [47].
  - The mu wave is even suppressed when observing another person performing a motor or abstract motion with biological characteristics. Researchers such as V. S. Ramachandran and colleagues have suggested that this is a sign that the mirror neuron system is involved in mu wave suppression, [48] although others disagree.
- *Beta wave*: 13–25/12.5–30 Hz (Alertness). This band is divided into three sub-bands: Low Beta Waves (12.5–16 Hz, “Beta 1 power”), Beta Waves (16.5–20 Hz, “Beta 2 power”), and High Beta Waves (20.5–28 Hz, “Beta 3 power”) [49].
- *Gamma wave*: >25/25–140 Hz (Awareness). They correlate with large-scale brain network activity and cognitive phenomena, such as working memory, attention and perceptual grouping [50].

Table 1 summarizes the mental activities with their respective EEG signal frequency bands.

Motor cortical activity measured with EEG-BCI systems is most evident in Alpha(α) and Beta(β) frequency bands, corresponding to 7–13 Hz and 13–30 Hz, respectively [42]. Power Spectral Density (PSD) typically measures features that determine the movement intention in the frequency ranges of α and β [51,52,53]. Yong Zhang et al. [52] used Wavelet coefficients as characteristics for the classification of mental tasks, contributing to the accuracy of the temporal resolution in the algorithm. In addition, several methods apply time series analysis to EEG signals, such as wavelet coherency analysis, continuous wavelet transform, empirical wavelet transform, and empirical mode decomposition [54,55].

Some ML algorithms (supervised and unsupervised learning) used for detecting movement intentions for the upper limbs are: Support Vector Machines (SVMs) [51,56], Artificial Neural Networks (ANNs) [51], Linear Discriminant Analysis (LDA) [51,53], and clustering algorithms [38,40]. SVMs perform better when detecting movement intentions for the upper limbs [51,56].

### 3.3. Brain Areas

The Penfield homunculus is a map of the cerebral cortex that shows that specific human brain areas are dedicated to processing the motor and sensory functions of each part of the body. For example, the lower and upper limbs are linked to certain motor and somatosensory cortex areas. In addition, each limb is associated with the contralateral side of the brain, i.e., the right cerebral hemisphere controls the motor activity of the left side of the body and vice versa. Thus, if the brain receives a stimulus in a specific part of the cerebral cortex, the body part linked to that area of the brain will be activated. [57].

The somatosensory cortex processes and treats sensory information from the dermis, muscles, and joints and performs voluntary hand movements [58]. On the other hand, the motor cortex plans, controls, and executes all voluntary motor actions.

The cerebral cortex is divided into areas that react to the stimuli in the organism and coordinate body functions. These areas are known as Brodmann areas, defined and numbered by the German anatomist Korbinian Brodmann in 1909. [25].

**Figure 2:**
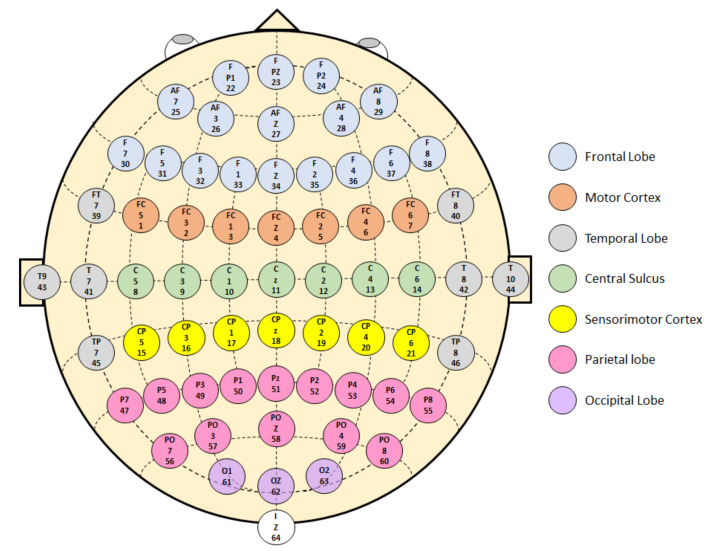
Brodmann areas in the 10-10 electrode positioning system.

The American Electroencephalographic Society standardized the international 10-10 system with 64 electrodes to ensure throughput and replicability in EEG research. The 10-10 refers to the actual distances between adjacent electrodes, 10% of the central sagittal curve or the central coronal curve, as shown in Figure 2. In the diagram, each letter refers to individual brain regions [38]: Frontal Coronal (F), Fronto-Central/Temporal (FC/FT), Temporo-/Central-Parietal (TP/CP), Parietal Coronal (P), Anterior-Frontal Coronal (AF), Parieto-Occipital Coronal (PO) and Occipital (O). Furthermore, Figure 2 shows the 10-10 system of electrode placement corresponding to Brodmann’s areas. The colors represent the function of each area: motor, somatosensory, attention, visual, executive, memory, emotion regulation, and sound.

## 4. Research Methodology

### 4.1. Scope and Research Questions

The review conducted in this paper is an overview according to the classification given in [28]. It aims to present a survey of specialized scientific papers about the detection of lower-limb movement intentions using BCI/EEG-based approaches. It focuses on studies that followed the motor imagery paradigm, as defined in [29]. At a signal analysis level, for the sake of a subsequent interpretability, PR-driven approaches are of interest for this work, with particular emphasis on the PR stages such as preprocessing, feature extraction, and classification. Along with all of the above, the experimental methodologies for data acquisition are also surveyed. Specifically, the following questions are addressed:

- What are the experimental methodologies used during data acquisition?
- What is the preprocessing technique used on EEG signals?
- What are the techniques used in feature extraction?
- What are the classification algorithms used in the detection of lower-limb movement intentions?

### 4.2. Search Method

**Figure 3:**
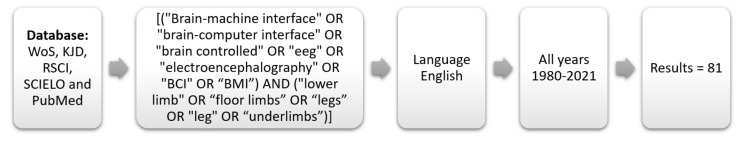
Search Results on WoS, KJD, RSCI, SCIELO, and PubMed.

According to the scope presented in Section 4.1, on 20 July 2021, an extensive but specialized literature search was conducted in: the Web of Science (WoS) core collection (2001–present), KCI—Korean Journal Database (1980–present), RSCI—Russian Science Citation Index (2005–present), SciELO Citation Index (2002–present), and PubMed. The search covered studies published between 1980 and 2021. The combinations of search terms were: ((“Brain-machine interface” OR “brain–computer interface” OR “brain controlled” OR “eeg” OR “electroencephalography” OR “BCI” OR “BMI”) AND (“lower limb” OR “floor limbs” OR “legs” OR “leg” OR “underlimbs”)). After searching, the first filter was to consider only full-text reports published in English. This search resulted in 81 scientific papers, as shown in Figure 3.

**Figure 4:**
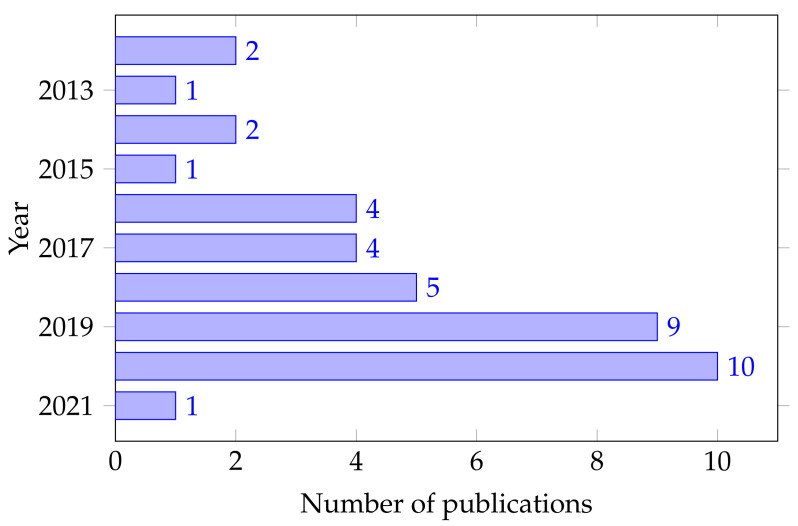
Publications per year about EEG signal classification during lower limbs tasks.

Figure 4 shows that 50% of the 81 papers resulting from the search query were published in the last ten years. This growth indicates an increasing interest in researching this topic. Moreover, the distribution of publications shows the increase in research papers over time, e.g., four papers in 2017, five papers in 2018, and twice as many between 2019 and 2020. This growth of 24% in the number of publications in the last three years alone indicates the relevance of this research topic.

We observe that 59% of the papers were published in Neurosciences/Neurology and 25% in Engineering, Computer Science, and Science and Technology. The remaining 16% belongs to Physiology, Anatomy Morphology, Psychology, and Computational Biology. These percentages show a growth in the close collaboration between technical areas and neuroscience.

**Figure 5:**
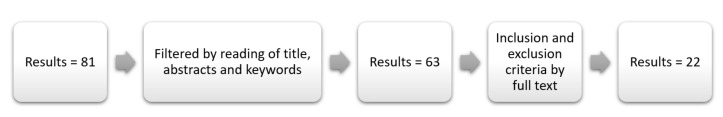
Data filtering process and inclusion/exclusion criteria.

Within the 81 papers, we defined the following search terms: Reading title, abstract, and keywords linked to human-related studies. As a result, we obtained 63 papers. Subsequently, based on the complete reading of the 63 papers, we applied the following inclusion and exclusion criteria: exclude duplicity, exclude papers that used steady-state visual evoked potential (SSVEP) to detect motor intentions, and include documents related to EEG signal processing while performing lower limb tasks and motor imagery tasks. However, we also chose to include papers studying both upper and lower limbs, not just lower limbs. As a result, 46 papers were excluded and 5 papers were included for cross-reference; thus, we were left with 22 papers for this research, as shown in Figure 5.

The 22 resulting papers mainly focus on analyzing EEG signals acquired noninvasively during lower limb motor and motor imagery tasks. Figure 2 shows that the lower limbs are linked to certain motor and somatosensory cortical areas, while the upper limbs are linked to other motor and somatosensory cortical areas. Therefore, physiologically, recording EEG signals while performing upper limb tasks will differ from lower limb tasks. This study explores the data acquisition methodologies, the feature extraction algorithms, and the classification algorithms used for capturing and interpreting EEG signals during lower limb tasks.

### 4.3. Data Extraction

We extracted general characteristics from the selected research papers, including the method employed in preprocessing the EEG signals, the number of volunteers recruited, the type of EEG signal used, whether a modality such as EMG, EOG, or other mechanical sensor was involved, and the main findings. We then performed individual abstract evaluations to determine which papers might meet inclusion considerations. For papers that met the inclusion criteria, we obtained the full-text content. The documents were then categorized according to the type of exoskeleton, either upper limb or lower limb. Nevertheless, we included papers dealing with both upper and lower limbs and not just lower limbs.

## 5. Results

As a result of the systematic review, we finally chose 22 papers about EEG-based monitoring for detecting lower-limb movement intentions, as listed in Table 2.

### 5.1. Experimental Methodology

**Figure 6:**
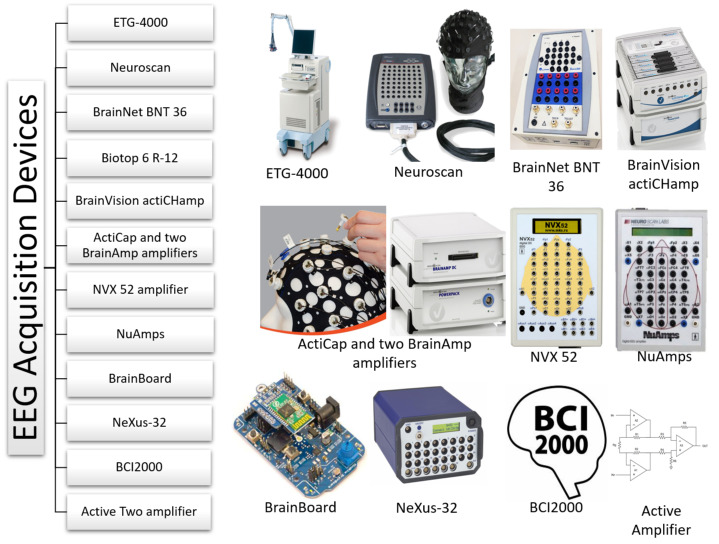
EEG Acquisition Devices used in reviewed works. Associated bibliographic references: ETG-4000 [60], Neuroscan [62,63,65,70,76,80], BrainNet BNT 36 [68,69], Biotop 6 R-12 [73], BrainVision actiCHamp [74], ActiCap and two BrainAmp amplifiers [75], NVX 52 amplifier [71], NuAmps [72], BrainBoard [77], NeXus-32 [78], BCI2000 [79], and Active Two amplifier [59,66]. The images displayed here are freely available from the manufacturer/provider’s official website and are exclusively used for non-profit, academic purposes. ETG-400: https://www.usa.philips.com/healthcare/resources/landing/fnirs; Neuroscan, NuAmps: https://compumedicsneuroscan.com; BrainNet BNT 36: https://www.emsamed.com.br; BrainVision actiCHamp, ActiCap and two BrainAmp amplifiers: https://brainvision.com; NVX 52 amplifier: https://mks.ru; BrainBoard: https://github.com/gskelly/eeg; Nexus-32: https://www.biofeedback-tech.com; BCI2000: https://www.bci2000.org. Last accessed (all of them): 10 February 2022).

Figure 6 shows the devices used by the authors for recording EEG signals. There is commercial equipment such as: ETG-4000 [60], Neuroscan [62,63,70,76], BrainNet BNT 36 [68,69], Biotop 6 R-12 [73], BrainVision actiCHamp [74], ActiCap and two BrainAmp amplifiers (Brain Products GmbH) [75], NVX 52 amplifier [71], NuAmps [72], BrainBoard using an ADS-1299 chip module (an open-source EEG hardware platform) [77], NeXus-32 bioamplifiers [78], and BCI2000 [79]. Other authors have used combined equipment such as Two 32-channel EEG amplifiers (Synamps, Neuroscan) [65]. In addition, one of the researchers used part of the FP1 electrodes of the Nuamps Express Neuroscan to record electrooculography (EOG) [80]. Finally, other authors have used the Active Two amplifier [59,66].

Based on Table 1 and Table 2, we can classify the experimental methodology proposed by the authors into the following types:

- Active Movements
  - In their work on cortical activity tracking, Gwin, J. T., and Ferris, D.P. [59] recorded EEG signals in the 8–30 Hz frequency band from eight healthy right-handed subjects (seven men and one woman) between 21 and 31 years old. The volunteers were seated and performed movements using a knee device that assisted involuntary movements during the experiment. The tasks they performed were: isometric and isotonic ankle and knee movements. The physical tasks were performed with the dominant limb. As additional sensors, Gwin and Ferris used a load cell to measure the force and a goniometer to measure the flexion angle. No visual or audible stimulation was used to indicate to the volunteers when to execute the movement.
  - Chou et al. [67] recorded EEG signals from five volunteers with a Spinal Cord Injury (SCI). The volunteers stood facing a monitor during the experiment, and an avatar told them when to perform the movement. With the help of an exoskeleton, they performed left and right stepping movements. No additional sensors were used.
  - Chang et al. [72] recorded EEG signals in the 0.5–25 Hz frequency band from three healthy volunteers in the first experiment and two post-stroke patients in the second (two trials, one with and one without the music rehabilitation system). The volunteers were standing during the experiment and used mixed and augmented reality as a visual stimulus to indicate when to execute the movement. The task performed was walking. Motion capture sensors (Notch-knee joint angle) were used as additional sensors to obtain the knee flexion angle of the volunteers.
  - Hoshino et al. [73] recorded EEG signals in the alpha (8–12 Hz), beta (13–30 Hz), low-beta (13–19 Hz), and high-beta (20–30 Hz) frequency band from 24 post-stroke patients. Patient selection criteria were: first-ever stroke (ischemic or hemorrhagic), supratentorial lesion, between 20 and 85 years old, independently active before the stroke, and right hand dominant. They included patients within four weeks of the event who did not lose all of their motor function. As a result, 24 participants with an average age of 62 years were chosen. Patients were lying on a bed with their eyes closed during the experiment. The tasks performed were ankle movements, dorsiflexion, and plantar flexion. No additional sensors were used. No visual or audible stimulation was used to indicate to the patients when to perform the movement.
  - Choi et al. [74] recorded EEG signals in the 7–34 Hz frequency band from 10 healthy volunteers. All volunteers were right-handed males with an average age of 26.6 years and no history of neurological disorders. The volunteers performed active movements, and the task was gait and sit. Visual stimulation was used to indicate to the patients when to execute the movement. No additional sensors were used.
- Motor Imagery
  - Tariq et al. [61] recorded Event-Related Desynchronization and Event-Related Synchronization (ERD/ERS) EEG signals from 14 healthy volunteers. Participants were seated during the experiment and performed motor imagery tasks. No additional sensors were used. A monitor was used as visual stimulation.
  - Hsu et al. [63] recorded EEG signals in the 8–30 Hz frequency band from eight healthy volunteers aged 20–25 years. The tasks performed by the volunteers were left and right stepping. Electrooculography (EOG) was used as an additional sensor because a screen was used for visual stimulation.
  - Al-Quraishi et al. [64] recorded Event-Related Desynchronization (ERD) EEG signals from three healthy volunteers and four Spinal Cord Injury (SCI) patients. The participants were seated during the experiment and performed motor imagery with the aid of a prosthetic knee. The task performed was walking and idling. A screen was used for visual stimulation. No additional sensors were used during the experiment.
  - In their work on implementing a BCI system, Gu et al. [70] recorded EEG signals in the 1–30 Hz frequency band from 11 healthy right-handed volunteers (4 males and 7 females) aged 22–27 years with no history of neuromuscular disorders. The subjects were seated during the experiment and performed motor imagery. The task performed was foot dorsiflexing. Vertical and horizontal electrooculography (EOG) was used as an additional sensor. A screen was used as a means of visual stimulation to perform the timed tasks.
  - Ortiz et al. [75] recorded EEG signals in the 2–60 Hz frequency band from three adult volunteers without physical impairments. Participants were seated during the experiment and performed motor imagery. The task performed was walking. No additional sensors were used. Auditory stimulation was used to indicate the execution of the task while the participant was thinking about the action.
  - Do et al. [78] recorded EEG signals at a sampling rate of 256 Hz from two subjects (one able-bodied and one with paraplegia due to Spinal Cord Injury (SCI)). The task performed was kinesthetic motor imagery (KMI). The task consisted of walking using BCI-edRoGO along a linear trajectory. Electromyography (EMG) signals were measured to rule out BCI control by voluntary leg movements in the healthy subject.
- Motor imagery—Active Movements
  - Gordleeva et al. [71] recorded EEG signals in the 8–15 Hz frequency band from eight healthy volunteers aged 20–27 years. EEG and EMG signals to perform a leg lift movement were obtained using an HMI. The tasks performed were motor imagery and active movement. EMG sensors were also used for feedback of the lower limb exoskeleton control system.
  - Kline et al. [76] recorded EEG signals in the 8–45 Hz frequency band from sixteen healthy male volunteers with an average age of 24.7 years. EEG and fMRI data were collected during executed and imagined movements of the lower limbs. The tasks performed were motor imagery and active movement. Participants observed the Computer-Generated Image (CGI) of a walking human being and performed a lower limb movement or imagined it following the CGI rhythm.
  - Murphy et al. [77] recorded EEG signals in the 1–100 Hz frequency band from a 36-year-old male that underwent a right transfemoral amputation. Two additional Gyro + Accelerometer sensors were used. The subject performed ten visits of two test sessions using a lower limb prosthesis. A conductive gel was used to fill the space between the electrodes and the scalp to ensure good conductivity and minimize noise artifacts. At the first visit, the subject was trained to use the BCI system to control a switch on a lower limb prosthesis. Each training visit had two sessions. In the first session, training ensued. EEG signals were recorded while the subject performed motor imagery tasks of the amputated limb. These data were used to determine the parameters needed to predict movement intention. In the second session, these parameters were used to control a knee locking mechanism in the prosthesis in real-time while walking on parallel bars. No additional sensors were used. Auditory stimulation was used to indicate the execution of the task while the participant was thinking about the action.
  - Asanza et al. [79] used a database of 64-channel EEG signals recorded using the so-called BCI2000 system. Both the acquisition system and the data are widely described in [81]. EEG signals were recorded at 160 samples per second from eight healthy subjects. The tasks used for this study were motor activity and motor imagery of dorsi and plantar flexion of both feet. No additional sensors were used.
- Motor imagery—Active Movements—Attempted movements
  - Jochumsen et al. [80] recorded EEG signals in the 0.05–10 Hz frequency band from twelve healthy subjects (two females and ten males: 28 ± 4 years old) and six stroke patients with lower limb paresis. The subject was seated in a comfortable chair with the right foot (or the affected foot) attached to a foot pedal where a force transducer was set up. The tasks performed were executed and attempted movements and motor imagery kinetics. The healthy subjects performed the two tasks with Motor Execution (ME) and Motor Imagery (MI), while the stroke patients were asked to attempt the movements.
- Movement intention—Active Movements
  - Rea et al. [60] recorded EEG signals from seven right-handed patients (four men and three women) with chronic stroke and an average age of 54.7 years. The requirements for participation in the study were: interval since the stroke of at least 12 months, no psychiatric or neurological condition other than stroke, no cerebellar lesion or bilateral motor deficit, and ability to understand and follow instructions. The subjects were seated during the experiment and performed movements with a foot pedal. The tasks performed were hip movements with a knee and ankle constraint. The authors employed additional EMG sensors during the tasks.
  - Liu et al. [66] recorded EEG signals in the 0.1–1 Hz and 0.05–2 Hz frequency bands from ten healthy volunteers (seven males and three females) with an average age of 26.1 years. The subjects used a customize leg press as a gait trainer during the experiment. EMG sensors and a force pedal were used. In addition, an EOG sensor was employed as the subjects were in front of a monitor with visual stimulation to indicate the execution of plantar flexion.
  - Delisle-Rodriguez et al. [68] and Gurve, D. et al. [69] used the same data. They recorded EEG signals in the 8–24 Hz [68] and 0.1–30 Hz [69] frequency bands from ten healthy volunteers (three women and seven men) between 21 and 36 years old. The volunteers had to perform motor imagery and active movement. The task performed by the volunteers was to think about pedaling for five seconds and then actually pedal. sEMG signals were captured to verify the absence of muscle contractions. A screen with visual stimulation was used to perform the series of pedaling and gait movements [68,69].
- Assisted movements
  - Qiu et al. [62] recorded Event-Related Desynchronization (ERD) EEG signals from 12 healthy volunteers (five women and seven men) aged 20–26 years and a 56-year-old stroke patient with hemiplegia. The requirements for enrollment were: a minimum of 2.5 years since the last stroke, severe hemiparesis, and difficulty in extending the right knee. The tasks performed were right-leg lifts. A screen with visual stimulation was used to perform the series of movements. No additional sensors were used.
- Electrical lower limb stimulation
  - Hauck et al. [65] recorded EEG signals from six healthy right-handed volunteers with an average age of 24.5 years. In addition, Magnetic Resonance Imaging (MRI) was obtained from five volunteers for data recording. Subjects were lying down, and low amperage electrical stimulation was applied to the peroneal, proximal tibial, and distal tibial nerves. Electrooculography (EOG) sensors were also used.

**Figure 7:**
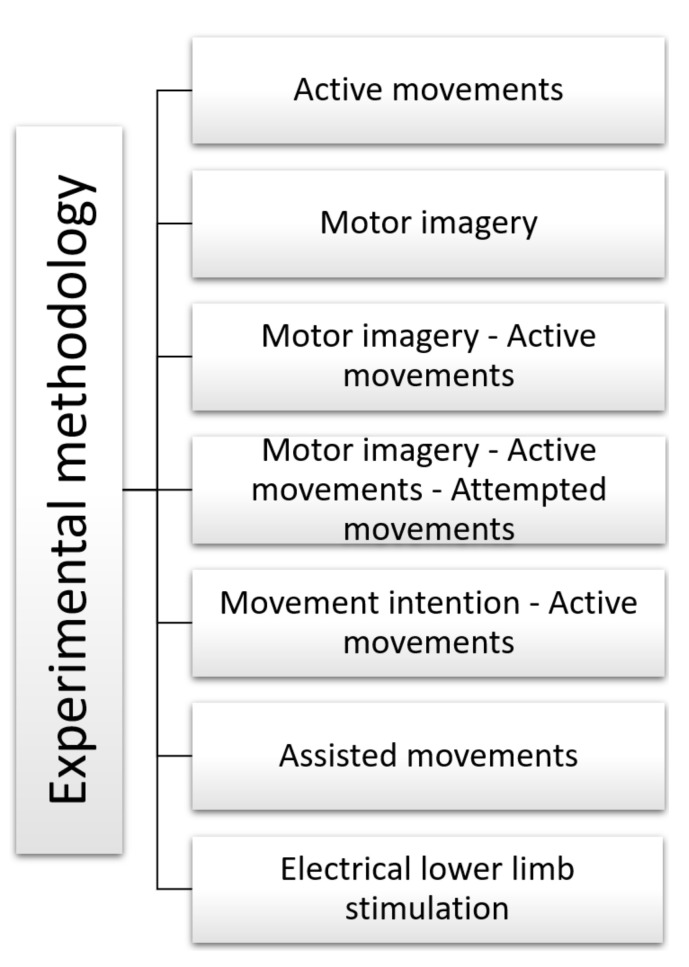
High-level taxonomy and related works for the experimental methodologies for data acquisition. Associated bibliographic references: active movements [59,67,72,73,74], motor imagery [61,63,64,70,75,78], motor imagery–active movements [71,76,77,79], motor imagery–active movements–attempted movements [80], movement intention–active movements [60,66,68,69], assisted movements [62], and electrical lower limb stimulation [65].

Figure 7 summarizes all experimental methodologies used to record EEG signals for lower limbs.

### 5.2. Data Preprocessing

Preprocessing techniques are essential as they help reduce noise in EEG signals. The techniques used for preparing the lower-limb EEG signals in the reviewed papers are:

- Butterworth filter
  - Gwin and Ferris [59] used a Butterworth 1 Hz High-Pass filter to remove noise from active movement EEG signals. Channels with a standard deviation greater than or equal to 1 mV were removed; channels whose kurtosis was higher than three standard deviations from the mean were removed; uncorrelated channels (r≤ 0.4) with nearby channels for more than 0.1% of the time-samples were removed.
  - Liu et al. [66] removed noise from EEG signals of movement intention and active movement using a sixth-order non-causal Butterworth filter for the 30–300 Hz frequency bands. In addition, they used Teager-Kaiser Energy Operator (TKEO) to condition signals, minimize background noise, and reduce movement artifacts. Conditioning also included 2nd order non-causal Low pass Butterworth filter 50 Hz.
  - Gurve et al. [69] eliminated noise from EEG signals of motor imagery and active movement using a second-order Butterworth filter from 0.1 to 30 Hz and Riemann geometry Non-negative Matrix Factorization (NMF).
  - Asanza et al. [79] eliminated noise from EEG signals of motor activity and imaginary motor using a two hundred-order Butterworth Infinite Impulse Response (IIR) filter from 8 to 30 Hz.
- Low-pass filter
  - Rea et al. [60] eliminated noise from EEG signals of movement intention and active movement using a low-pass filter Wavelet-minimum description length Gaussian low-pass filter with 4-second Fullwidth-Half-Maximum (FWHM).
  - Hauck et al. [65] used a low-pass filter below 100 Hz to remove noise from EEG signals of induced movements.
- Notch filter
  - Qiu et al. [62] used a notch filter at 50 Hz and Downsampling at 200 Hz to remove noise from EEG signals of movement intention and active movement.
  - Delisle-Rodriguez et al. [68] removed noise from EEG signals of movement intention and active movement using a notch filter at 60 Hz, Spectrogram based on Short-Time Fourier Transform (SSTFT), and Riemann geometry.
  - Ortiz et al. [75] removed EEG signal noise from movement intention and active movement using a notch filter at 60 Hz.
- Band-pass Filter
  - Hsu et al. [63] removed noise from EEG signals of movement intention and active movement using a band-pass filter in 4–40 Hz frequency bands.
  - Gu et al. [70] removed noise from EEG signals of movement intention and active movement using a band-pass filter in 1–30 Hz frequency bands and Independent Component Analysis (ICA).
  - Gordleeva et al. [71] used a band-pass filter in 8–15 Hz frequency bands to remove noise from EEG signals of movement intention and active movement.
  - Chang et al. [72] used a band-pass filter in 1–50 Hz frequency bands to remove noise from EEG signals of movement intention and active movement.
  - Hoshino et al. [73] removed noise from EEG signals of movement intention and active movement using a band-pass filter in the 0.5–100 Hz frequency bands and multiple linear regression analysis.
  - Kline et al. [76] removed EEG signal noise from DC offset and noise associated with blinking using a bandpass filter between 5 and 55 Hz with a roll-off of 20 dB/decade.
  - Murphy et al. [77] used non-stimulus BCI signal event-related desynchronization EEG (ERD). For this, he employs a bandpass filter for the beta band frequencies (1–100 Hz) along with a custom-made MATLAB toolbox (BCI2VR).
- Spatial Filter
  - Choi et al. [74] removed EEG signal noise from movement intention and active movement using a Filter Bank Common Spatial Pattern (FBCSP).
  - Jochumsen et al. [80] used an Optimized Spatial Filter (OSF). The output of the OSF (one channel) was bandpass filtered from 0.05 to 10 Hz with a second-order Butterworth filter and downsampled to 20 Hz.

**Figure 8:**
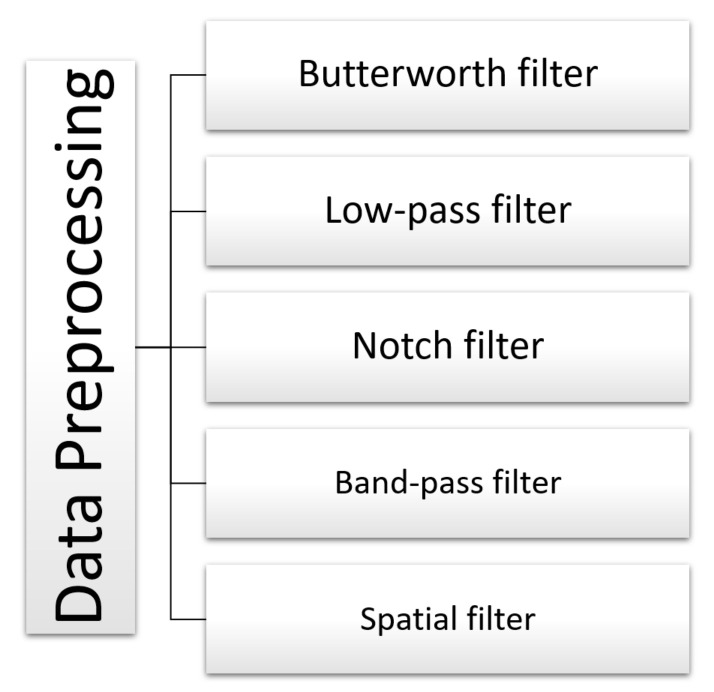
High-level taxonomy and related works for data preprocessing. Associated bibliographic references: butterworth filter [59,66,69,79], low-pass filter [60,65], notch filter [62,68,75], band-pass filter [63,70,71,72,73,76,77], and spatial filter [74,80].

Figure 8 summarizes all preprocessing techniques used on the EEG signals.

### 5.3. Feature Extraction

The following is a summary of the feature extraction techniques:

- Time-domain
  - Jochumsen et al. [80] used six time-domain features extracted from the 2-second data segment before movement detection. The features were: (i+ii) slope and intercept of a linear regression of the entire data segment, (iii+iv) slope and intercept of a linear regression of the data segment from the point of detection and 0.5 s prior to this point, (v) average amplitude of the entire data segment, and (vi) the peak of maximum negativity.
- Based on ERD/ERS
  - Qiu et al. [62] used Event-Related Spectral Perturbation (ERSP) and Event-Related Desynchronization (ERD) for feature extraction from highly event-related EEG signals in right leg lifting tasks.
  - Murphy et al. [77] used Event-Related Desynchronization (ERD) for feature extraction from the beta band (16–24 Hz). It was calculated in real-time against baseline activity when the subject was relaxed.
- Based on Filter bank
  - Hsu et al. [63] used the Filter-bank CSP (FB-CSP) for feature extraction from highly event-related EEG signals in left-and-right stepping and motor imagery tasks.
  - Gordleeva et al. [71] used the Common Spatial Pattern Filter (CSP) for feature extraction from highly event-related EEG signal characteristics in motor imagery and active movement leg lifting tasks.
- Based on Power Analysis
  - Rea et al. [60] used T-value for feature extraction of EEG signals with high temporal resolution in movement intention and active movement tasks of hip movements with a knee and ankle constraint.
  - Hauck et al. [65] used the mean global field power signal-to-noise ratio (SNR) for feature extraction of EEG signals of induced movements.
  - Liu et al. [66] used Gini index scores of tree nodes for feature extraction of EEG signals related to movement intention and active movement.
  - Chang et al. [72] used the Power Spectrum over the main channel for feature extraction of EEG signals of walking active movement.
  - Ortiz et al. [75] used Empirical Mode Decomposition (EMD) for Intrinsic Mode Functions (IMFs) and Variation of Power for IMFs for feature extraction of EEG signals from motor imagery of walking.
  - Kline et al. [76] used the power spectrum value of all the studied frequencies (alpha, beta, and gamma) for each EEG electrode.
  - Do et al. [78] used spatio-spectral features from the 8–10 Hz frequency band for able-bodied subjects and the 10–12 Hz frequency band for SCI subjects.
  - Asanza et al. [79] used Power Spectral Density (PSD) from 8 to 30 Hz, calculated at 10 s of sampling of each EEG electrode.
- Based on Correlation Analysis
  - Gwin and Ferris [59] used an Adaptive Mixture Independent Component Analysis (AMICA) for feature extraction of EEG signals with a high temporal resolution in isometric and isotonic ankle and knee movements.
  - Delisle-Rodriguez et al. [68] and Gurve, D. et al. [69] used a Neighborhood Component Feature Selection (NCFS) algorithm for feature extraction of EEG signals from motor imagery and active movement during the pedaling task.
  - Gu et al. [70] used Sparse Multinomial Logistic Regression for feature extraction of EEG signals from motor imagery during the foot dorsiflexing task.
  - Hoshino et al. [73] used Amplitude Envelope Correlation (AEC) for feature extraction of EEG signals from active movement during ankle movements, dorsiflexion, and plantarflexion.
  - Choi et al. [74] used Mutual Information-based Best Individual Feature (MIBIF) for feature extraction of EEG signals from active movement during the gait and sit task.

**Figure 9:**
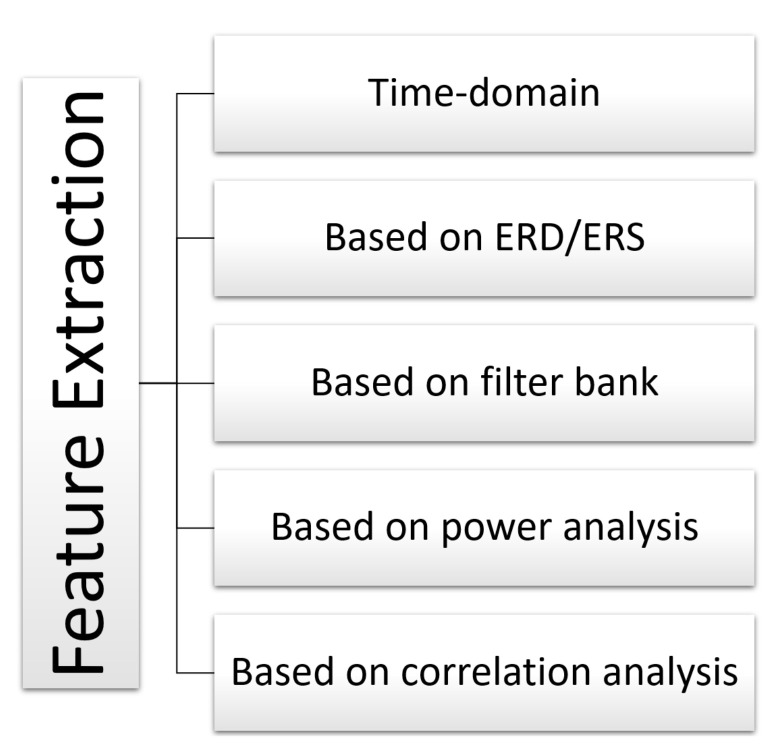
High-level taxonomy and related works for feature extraction. Associated bibliographic references: time-domain [80], based on ERD/ERS [62,77], based on filter bank [63,71], based on power analysis [60,65,66,72,75,76,78,79], and based on correlation analysis [59,68,69,70,73,74].

Figure 9 summarizes all the feature extraction techniques used on the recorded EEG signals.

### 5.4. Classification Algorithms

The following is a summary of the classification algorithms used:

- Bayesian Classifier
  - Gwin and Ferris [59] used a four-way linear naïve Bayesian Classifier to classify isometric and isotonic ankle and knee movements with an accuracy of 87%.
  - Do et al. [78] used a Bayesian Classifier, performing stratified 10-fold cross-validation and used 90% of the EEG data to train. This offline analysis resulted in a model classification accuracy of 94.8 ± 0.8% and 77.8 ± 2.0% for able-bodied and SCI subjects, respectively.
- Support Vector Machine (SVM)
  - Hsu et al. [63] used Fuzzy SVM to classify EEG signals in left-and-right stepping motor imagery tasks with an accuracy of 86.25%.
  - Gu et al. [70] used SVM to classify EEG signals in motor imagery during the foot dorsiflexing task with an accuracy of 67.13%.
  - Choi et al. [74] used SVM to classify the EEG signals of active movement during the gait and sit task, achieving 80% accuracy.
  Jochumsen et al. [80] classified EEG signals from executed, imaginary, and attempted movement tasks using SVM with accuracy of 57 ± 3%, 53 ± 6%, and 47 ± 7%, respectively.
- Liu et al. [66] used Random Forest to classify the EEG signals related to movement intention and active movement with an accuracy of 85%.
- Linear discriminant analysis (LDA)
  - Rea et al. [60] used LDA with linear kernel to classify the EEG signals in movement intention and active movement tasks of hip movements with a knee and ankle constraint. They achieved an accuracy of 67.77%.
  - Delisle-Rodriguez et al. [68] and Gurve, D. et al. [69] classified EEG signals from motor imagery and active movement during the pedaling task using LDA with accuracies of 92.85% and 96.66%, respectively.
  - Gordleeva et al. [71] classified EEG signals in motor imagery and active movement leg lifting tasks with LDA and achieved an accuracy of 65.7%.
  - Murphy et al. [77] classified EEG signals from motor imagery with an offline LDA model. This model was made for online detection of the subject’s intention to activate the switch by imaging lower-limb movement. The algorithms used the BCI2VR toolbox.
- Neural Network (NN)
  - Kline et al. [76] used a neural network (NN) implemented in Python 3.7 to classify right and left lower limb movement. The model used the Keras toolbox, achieving greater than 66% accuracy.
  - Asanza et al. [79] used a neural network (NN) trained in Matlab and then implemented it on Field-Programmable Gate Arrays (FPGAs). The model classified motor activity and motor imagery of both feet, with accuracies of 92.1% and 93.8%, respectively.

**Figure 10:**
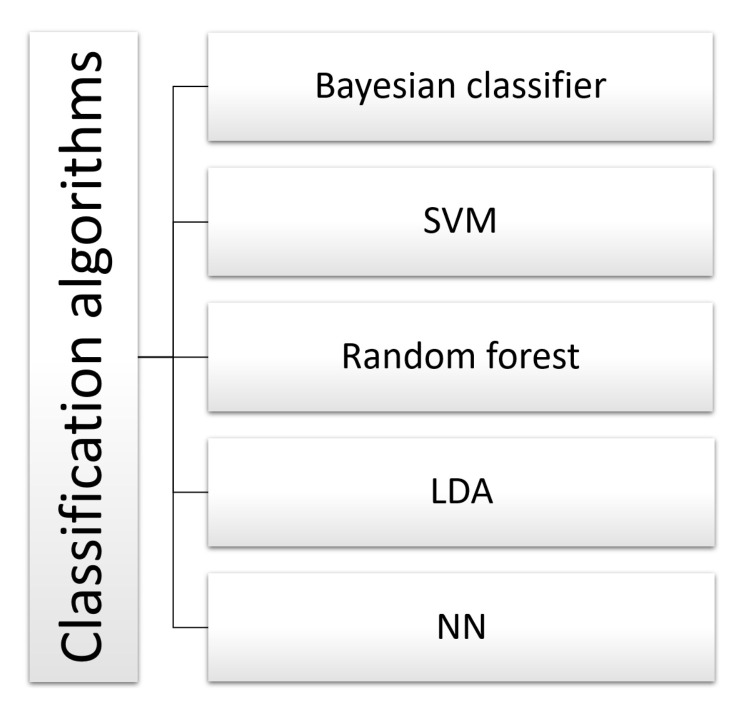
High-level taxonomy and related works for classification algorithms. Associated bibliographic references: bayesian classifier [59,78], SVM [63,70,74,80], random forest [66], LDA [60,68,69,71,77], and NN [76,79].

Figure 10 summarizes all the classification algorithms used on the recorded EEG signals.

## 6. Discussion

**Figure 11:**
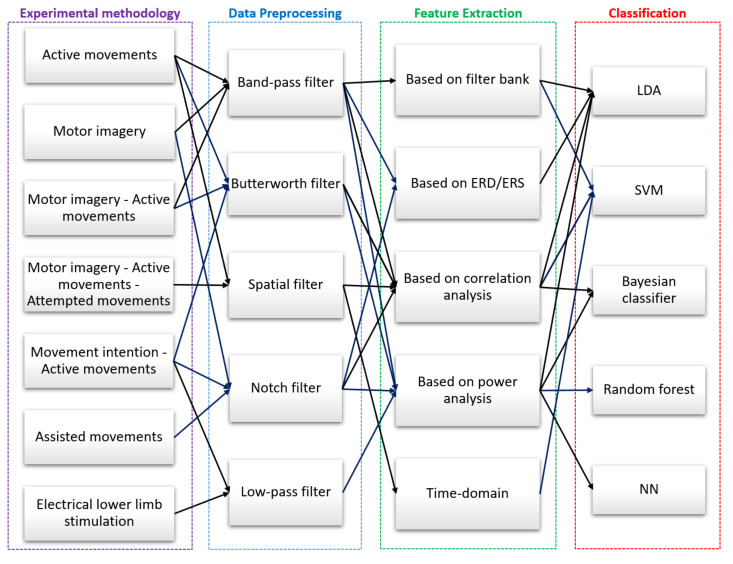
A joint flowchart summarizing the types of tasks and algorithms that have been used at each stage. Associated bibliographic references per task: Experimental methodology: active movements [59,67,72,73,74], motor imagery [61,63,64,70,75,78], motor imagery–active movements [71,76,77,79], motor imagery - active movements - attempted movements [80], movement intention–active movements [60,66,68,69], assisted movements [62], and electrical lower limb stimulation [65]; Data preprocessing: band-pass filter [63,70,71,72,73,76,77], butterworth filter [59,66,69,79], spatial filter [74,80], notch filter [62,68,75], and low-pass filter [60,65]; Feature extraction: based on filter bank [63,71], based on ERD/ERS [62,77], based on correlation analysis [59,68,69,70,73,74], based on power analysis [60,65,66,72,75,76,78,79], and time-domain [80]; Classification: LDA [60,68,69,71,77], SVM [63,70,74,80], bayesian classifier [59,78], random forest [66], and NN [76,79].

This section starts by discussing the experimental methodologies and the different types of tasks performed by the volunteers. Figure 11 summarizes the different tasks performed by the participants and the algorithms reported by the authors in the stages of preprocessing, feature extraction, and classification of EEG signals. These tasks involve the lower limbs, and during their execution, EEG signals from the cerebral cortex were recorded. In applications that require motor activity to control or test devices, volunteers performed tasks with voluntary or active movement [59,67,72,73,74]. In applications aimed at improving visual coordination and controlling prosthetic equipment, subjects performed motor imagery [61,63,64,70,75,77,78]. In prosthetic equipment control applications, volunteers combined motor imagery [71] and movement intentions [60,66,68,69,79] with active movement. Several works also used combined tasks such as executed and imaginary movements for healthy subjects and attempted movements for stroke patients [80]. Other applications use computer-generated images (CGI) of a person walking that participants followed to perform a lower limb task [76]. Finally, there are tasks related to non-voluntary movements such as induced movements [65] and assisted movements [62] for rehabilitation and motor coordination applications.

Regarding the preprocessing stage, we can determine that for EEG signals involving active movement, motor imagery, and the combination of both, Band-pass filters are primarily used in frequency ranges from 8 to 30Hz [63,70,71,72,73,77], capturing Alpha (α), Mu (μ), Beta (β) brainwaves, as well as gamma (γ) brainwaves, up to 55 Hz [76], as shown in Table 1. Moreover, several works reported the use of low-pass filters for frequencies below 100 Hz, thus eliminating noise from EEG signals [60,65]. Other authors removed power grid noise from EEG signals using a notch filter for 50 Hz [62] or 60Hz [68,75], depending on the location of the power grid used. On the other hand, in applications related to movement intention with active movement tasks, Butterworth filters are usually employed in frequency ranges from 0.1 to 30 Hz, capturing Delta (δ), Theta (θ), Alpha (α), Mu (μ), and Beta (β) brainwaves [59,66,69,79]. Finally, we also report papers that used a spatial filter for frequency bands from 0.05 to 10 Hz. An Optimized Spatial Filter (OSF) was used, and the output was bandpass filtered using a second-order Butterworth filter for executed, imaginary, and attempted movements [80]. A Filter Bank Common Spatial Pattern (FBCSP) was used for movement intention and active movement [74].

For the feature extraction stage, we determined that for EEG signals of active movement and motor imagery combined with active movement, they used feature extraction based on a filter bank [63,71]. For tasks such as executed and imaginary movements for healthy subjects and attempted movements for stroke patients, time-domain features were used [80]. Furthermore, some authors used feature extraction based on correlation analysis for motor tasks such as active movement, movement intention with active movement, and motor imagery [59,68,69,70,73,74]. On the other hand, other authors used feature extraction based on power analysis for tasks such as active movement, movement intention with active movement, induced movements, and motor imagery [60,65,66,72,75,76,78,79]. In addition, techniques based on ERD/ERS have been used in tasks such as assisted movements [62,77].

In the classification stage, we can say that the authors used SVM in motor imagery tasks, achieving an accuracy of 86.25%, 67.13%, and 52.3% (average) [63,70,80]. Several authors have also used NNs to classify left and right lower limb imagined movement, achieving a 66.6% accuracy [76]. In motor activity and motor imagery classification of both feet, the model achieved 92.1% and 93.8% accuracies, respectively [79]. In the classification of active movement, they reported an accuracy of 80% [74]. Classification algorithms based on Naive Bayesian, frequency bands’ power comparison has been used to classify active movement tasks, achieving an accuracy of 87% [59,73]. Other Bayesian classification algorithms were used to classify kinesthetic motor imagery (KMI), achieving 94.8 ± 0.8% and 77.8 ± 2.0% accuracies for able-bodied and SCI subjects, respectively, [78].

Some works have used LDA for tasks such as movement intention with active movement, reaching accuracies of 67.77%, 92.85%, 96.66%, and more than 90% [60,68,69,77], and motor imagery tasks with active movement, with an accuracy of 65.7% [71]. In movement intention tasks with active movement, the random forest classification algorithm was used, achieving an accuracy of 85% [66].

## 7. Conclusions

This work has taken place on the basis that, on one hand, there is not a great number of BCI- or EEG-based studies focused on lower-limb motor task identification, and, on the other hand, this topic has not been widely exploited within a conventional pattern recognition (PR) framework. This is an important aspect as PR provides substantial advantages in terms of interpretability and modularity. In this study, we consider a PR system [26] mainly composed by stages for data acquisition, preprocessing, feature extraction, and classification. We present a topical overview of specialized scientific papers focused on BCI-driven detection of lower-limb movement intentions.

Regarding the experimental methodologies, the following physical tasks were established to record EEG signals: for capturing signals from active movement, the volunteers performed isometric and isotonic ankle and knee movements [59]. For recording signals from motor imagery and active movement, the volunteers performed movements using a lower limb exoskeleton control system [71]. In motor imagery, data acquisition was performed during left-and-right stepping [63]. Finally, for capturing signals from movement intention and active movement, the tasks were to think about pedaling for a while and then actually to pedal [69].

The papers that reported the highest accuracy employed the following algorithms for preprocessing: Butterworth filter in the 8–30 Hz frequency range for classifying active movements tasks [59]; band-pass filter in the 8–15 Hz frequency range for motor imagery and active movement tasks [71]; band-pass filter for the 8–30 Hz frequencies in motor imagery tasks [63]; finally, Butterworth filter for the 0.1–30 Hz frequencies for movement intention and active movements tasks [69].

In the feature extraction stage, the papers with the highest accuracy employed the following algorithms: adaptive mixture independent component analysis (AMICA) for classifying active movement tasks [59]; Common Spatial Pattern Filter (CSP) for motor imagery and active movement tasks [71]; Filter-bank CSP (FB-CSP) in motor imagery tasks [63]; and finally, the Neighborhood Component Feature Selection (NCFS) algorithm for movement intention and active movement tasks [69].

Regarding the works that performed classification, we observed that in signal classification during active movement tasks, the Naive Bayesian Classifier achieved the highest accuracy at 87% [59]. For signal classification during kinesthetic motor imagery (KMI), the Bayesian Classifier achieved the highest accuracy of 94.8% [78]. In motor imagery and active movement tasks, LDA classifies motor intentions with an accuracy of 65.7% [71]. SVM classifies intentions in motor imagery tasks, with an accuracy of 86.25% [63]. Finally, the LDA algorithm is again the most accurate when classifying motor intentions of movement intention and active movement tasks, with a 96.66% mark [69]. Table 3 summarizes the best algorithms according to their accuracy in classifying signals during each task performed by the volunteers.

## Figures and Tables

**Table 1:** EEG Rhythms.

Band	Frequency (Hz)	Mental State
δ	<4	Infants or Adults during Deep Sleep.
θ	4–7	Youngsters and adults in stages of drowsiness.
α	8–12	Young people and adults during low brain activity or rest.
μ	7.5–12.5	Present in the motor cortex during the execution or thinking of motor activities.
β	16–31	Present during active or busy thinking, state of concentration, high alertness, and anxiety.
γ	32	High Brain Activity.

**Table 2:** EEG-based control for lower limb movements.

Author [Ref]	Application	Subjects	Protocol	Task	EEG Signal	Other Input Signal
Gwin and Ferris [59]	Tracking cortical activity	8 healthy subjects	Active Movements	Isometric and isotonic ankle and knee movement	8–30 Hz	Load Cell
Rea et al. [60]	Custom pedal chair	7 chronic stroke patients	Movemenet intention—Active movement	Hip movements—knee and ankle constrained	-	EMG
Tariq et al. [61]	RE lower limb Exoskeleton	14 healthy subject	Motor imagery	Gait	ERD/ERS	-
Qiu et al. [62]	Personal assistance, VitalStim Therapy + visual coordination	12 healthy subject + 1 hemiplegic stroke patient	Active, assisted, and FES-induced movements	Right leg raise	ERD	-
Hsu et al. [63]	Elevated platform + visual coordination	8 healthy subject	Motor imagery	Left and right Stepping	8–30 Hz	EOG
Al-Quraishi et al. [64]	Prosthetic Knee	3 healthy and 4 SCI patients	Motor imagery	Walking and Idling	ERD	-
Hauck et al. [65]	sensory stimulation	6 healthy subject	Electrical lower-limb stimulation	-	-	MRI/EOG
Liu et al. [66]	Customize leg Press—Gait trainer + visual coordination	10 healthy subject	Movemenet intention—Active movement	Plantar flexion	(0.1–1 Hz) (0.05–2 Hz)	EOG, EMG, force on pedal
Chou et al. [67]	Avatar, BWS and Overground exoskeleton	5 SCI subjects	Motor execution	Left and right Stepping	-	-
Delisle-Rodriguez et al. [68]	Motorized pedal + visual coordination	10 healthy subject	Motor imagery—Active Movement	Pedaling	8–24 Hz	sEMG
Gurve et al. [69]	Motorized pedal + visual coordination	10 healthy subject	Motor imagery—Active Movement	Gait	0.1–30 Hz	sEMG
Gu et al. [70]	BCI system	11 healthy subject	Motor imagery	Foot dosiflexing	1–30 Hz	Vertical and horizontal EOG
Gordleeva et al. [71]	MI-based BCI lower limb exoskeleton control system	8 healthy subjects	Motor imagery—Active Movement	Leg lift	8–15 Hz	EMG
Chang et al. [72]	Mixed Augmented Reality (Hololens)	3 healthy subject + 2 stroke subjects	Active movements	Walking	0.5–25 Hz	Motion capture sensors (Notch—knee joint angle)
Hoshino et al. [73]	-	24 post-stroke subjects	Active movements	Ankle movements—Dorsiflexion and plantar flexion	alpha bands (8–12), beta (13–30), low beta (13–19), high beta (20–30)	-
Choi et al. [74]	MI-based BCI lower limb exoskeleton control system + visual coordination	10 healthy subject	Active movements	Gait and sit	7–34 Hz	-
Ortiz et al. [75]	MI-based BCI lower limb exoskeleton control system	3 healthy subject	Motor imagery	Walking	2–60 Hz	-
Kline et al. [76]	Mapping of spatial brain activity	16 healthy subjects	Executed and imagined	lower limb movements	alpha (8–12 Hz), beta (13–30 Hz) and gamma (31–45 Hz)	fMRI
Murphy et al. [77]	Event-related desynchronization (ERD) for lower extremity prosthesis control system	A subjects male suffered a right transfemoral amputation	Imaging right lower-limb movement and walking	Motor imagery task	16–24 Hz	Gyro + Accelerometer
Do et al. [78]	Brain-Controlled robotic gait orthosis	Two subjects (one able-bodied and one with paraplegia due to Spinal Cord Injury (SCI))	Complete a goal-oriented task of Walking along a linear path	Kinesthetic motor imagery (KMI)	8–10 Hz and 10–12 Hz	EMG electrodes and gyroscope
Asanza et al. [79]	BCI System	8 healthy subjects	Dorsi and plantar flexion of both feet	Motor activity and imaginary motor	8–30 Hz	-
Jochumsen et al. [80]	BCI for stroke rehabilitation	12 healthy subjects and 6 stroke patients with lower limb paresis	movement kinetics	Executed, imaginary, and attempted movements	0.1–10 Hz	Force transducer

**Table 3:** Classification accuracy of the algorithms for each type of task.

Task	Algorithm	Accuracy	Reference
Active Movements	Naive Bayesian Classifier	87%	[59]
Motor Imagery—Active Movements	LDA	65.7%	[71]
Motor Imagery	SVM	86.25%	[63]
Kinesthetic motor imagery (KMI)	Bayesian Classifier	94.8%	[78]
Movement Intention—Active Movements	LDA	96.66%	[69]
